# Supplementary material for: Discovery and application of insertion-deletion (INDEL) polymorphisms for QTL mapping of early life-history traits in Atlantic salmon
Source: BMC Genomics. 2010 Mar 8;11:156. doi: 10.1186/1471-2164-11-156 (PMC2838853; doi:10.1186/1471-2164-11-156)
Supplement: Additional file 2 — Information on developed 76 locus single-run INDEL panel in Atlantic salmon. Information on fluorescence labeling, primer concentrations, PCR pooling and links to alignments, INDEL motifs and GENESCAN (Burge and Karlin 1997) predictions of genes/exons are available in html format. [file 1471-2164-11-156-S2.ZIP › Additionalfile2/table20475.html]

Contig Alignment

```
Base	A B C D   
1	G . . .    
2	G . . .    
3	G . . .    
4	G . . .    
5	T . . .    
6	G . . .    
7	G . . .    
8	G . . .    
9	C . . .    
10	C . . .    
11	A . . .    
12	A . . .    
13	G . . .    
14	A . . .    
15	C . . .    
16	T . . .    
17	T . . .    
18	G . . .    
19	G . . .    
20	C . . .    
21	C . . .    
22	T . . .    
23	T . . .    
24	G . . .    
25	A . . .    
26	A . . .    
27	T . . .    
28	G . . .    
29	G . . .    
30	A . . .    
31	G . . .    
32	C . . .    
33	A . . .    
34	T . . .    
35	T . . .    
36	G . . .    
37	G . . .    
38	G . . .    
39	A . . .    
40	A . . .    
41	A . . .    
42	C . . .    
43	C . . .    
44	C . . .    
45	C . . .    
46	A . . .    
47	A . . .    
48	A . . .    
49	A . . .    
50	A . . .    
51	A . . .    
52	C . . .    
53	A . . .    
54	T . . .    
55	T . . .    
56	C . . .    
57	A . . .    
58	A . . .    
59	C . . .    
60	A . . .    
61	G . . .    
62	A . . .    
63	A . . .    
64	A . . .    
65	A . . .    
66	G . . .    
67	A . . .    
68	C . . .    
69	A A . .    
70	A A . .    
71	A A . .    
72	T T . .    
73	A A . .    
74	C C . .    
75	A A . .    
76	G G . .    
77	A A . .    
78	A A . .    
79	G G . .    
80	C C . .    
81	A A . .    
82	C C . .    
83	A A . .    
84	A A . .    
85	C C . .    
86	A A . .    
87	A A . .    
88	C C . .    
89	C C . .    
90	A A . .    
91	T T . .    
92	C C . .    
93	C C . .    
94	C C . .    
95	T T . .    
96	C C . .    
97	C C . .    
98	C C . .    
99	C C . .    
100	T T . .    
101	T T . .    
102	G G . .    
103	A A . .    
104	A A . .    
105	C C . .    
106	A A . .    
107	T T . .    
108	C C . .    
109	A A . .    
110	G G . .    
111	T T . .    
112	G G . .    
113	T T . .    
114	T T . .    
115	A A . .    
116	C C . .    
117	C C . .    
118	T T . .    
119	T T . .    
120	G G . .    
121	A A . .    
122	C C . .    
123	A A . .    
124	A A . .    
125	C C . .    
126	T T . .    
127	G G . .    
128	C C . .    
129	T T . .    
130	A A . .    
131	G G . .    
132	T T . .    
133	T T . .    
134	A A . .    
135	C C . .    
136	C C . .    
137	C C . .    
138	T T . .    
139	C C . .    
140	A A . .    
141	G G . .    
142	G G . .    
143	A A . .    
144	A A . .    
145	T T . .    
146	G G . .    
147	G G . .    
148	T T . .    
149	T T . .    
150	C C . .    
151	T T . .    
152	G G . .    
153	C C . .    
154	T T . .    
155	T T . .    
156	A A . .    
157	G G . .    
158	A A . .    
159	G G . .    
160	T T . .    
161	T T . .    
162	G G . .    
163	C C . .    
164	A A . .    
165	A A . .    
166	A A . .    
167	A A . .    
168	T T . .    
169	T T . .    
170	C C . .    
171	T T . .    
172	G G . .    
173	A A . .    
174	A A . .    
175	A A . .    
176	T T . .    
177	C C . .    
178	T T . .    
179	T T . .    
180	T T . .    
181	C C . .    
182	C C . .    
183	C C . .    
184	A A . .    
185	G G . .    
186	A A . .    
187	A A . .    
188	T T . .    
189	T T . .    
190	C C . .    
191	C C . .    
192	C C . .    
193	G G . .    
194	G G . .    
195	G G . .    
196	T T . .    
197	T T . .    
198	T T . .    
199	T T . .    
200	T T . .    
201	C C . .    
202	C C . .    
203	A A . .    
204	G G . .    
205	A A . .    
206	A A . .    
207	A A . .    
208	T T . .    
209	C C . .    
210	C C . .    
211	A A . .    
212	G G . .    
213	A A . .    
214	T T . .    
215	T T . .    
216	G G . .    
217	G G . .    
218	A A . .    
219	G G . .    
220	G G . .    
221	A A . .    
222	T T . .    
223	T T . .    
224	T T . .    
225	T T . .    
226	G G . .    
227	C C . .    
228	A A . .    
229	A A . .    
230	C C . .    
231	C C . .    
232	C C . .    
233	T T . .    
234	A A . .    
235	G G . .    
236	T T . .    
237	T T . .    
238	A A . .    
239	A A . .    
240	A A . .    
241	A A . .    
242	T T . .    
243	A A . .    
244	A A . .    
245	G G . .    
246	A A . .    
247	G G . .    
248	G G . .    
249	A A . .    
250	G G . .    
251	A A . .    
252	G G . .    
253	A A . .    
254	A A . .    
255	A A . .    
256	T T . .    
257	T T . .    
258	G G . .    
259	G G . .    
260	C C . .    
261	T T . .    
262	T T . .    
263	T T . .    
264	C C . .    
265	C C . .    
266	C C . .    
267	T T . .    
268	T T . .    
269	A A . .    
270	T T . .    
271	T T . .    
272	C C . .    
273	A A . .    
274	A A . .    
275	C C . .    
276	C C . .    
277	A A . .    
278	A A . .    
279	T T . .    
280	A A . .    
281	A A . .    
282	T T . .    
283	A A . .    
284	A A . .    
285	T T . .    
286	A A . .    
287	A A . .    
288	T T . .    
289	A A . .    
290	G G . .    
291	T T . .    
292	A A . .    
293	T T . .    
294	A A . .    
295	A A . .    
296	A A . .    
297	A A . .    
298	C C . .    
299	A A . .    
300	A A . .    
301	C C . .    
302	C C . .    
303	T T . .    
304	A A . .    
305	C C . .    
306	T T . .    
307	G G . .    
308	T T . .    
309	A A . .    
310	C C . .    
311	T T . .    
312	C C . .    
313	C C . .    
314	A A . .    
315	T T . .    
316	A A . .    
317	T T . .    
318	C C . .    
319	A A . .    
320	G G . .    
321	A A . .    
322	C C . .    
323	C C . .    
324	G G . .    
325	T T . .    
326	A A . .    
327	G G . .    
328	T T . .    
329	G G . .    
330	T T . .    
331	T T . .    
332	T T . .    
333	A A . .    
334	A A . .    
335	T T . .    
336	A A . .    
337	T T . .    
338	A A . .    
339	T T . .    
340	A A . .    
341	A A . .    
342	A A . .    
343	C C . .    
344	A A . .    
345	G G . .    
346	T T . .    
347	A A . .    
348	T T . .    
349	A A . .    
350	C C . .    
351	A A . .    
352	G G . .    
353	T T . .    
354	A A . .    
355	G G . .    
356	A A . .    
357	C C . .    
358	A A . .    
359	G G . .    
360	T T . .    
361	C C . .    
362	T T . .    
363	T T . .    
364	T T . .    
365	G G . .    
366	A A . .    
367	T T . .    
368	T T . .    
369	T T . .    
370	G G . .    
371	A A . .    
372	T T . .    
373	G G . .    
374	T T . .    
375	T T . .    
376	T T . .    
377	T T . .    
378	G G . .    
379	T T . .    
380	T T . .    
381	G G . .    
382	A A . .    
383	T T . .    
384	A A . .    
385	T T . .    
386	A A . .    
387	T T . .    
388	G G . .    
389	G G . .    
390	T T . .    
391	T T . .    
392	C C . .    
393	T T . .    
394	T T . .    
395	A A . .    
396	G G . .    
397	C C . .    
398	C C . .    
399	C C . .    
400	T T . .    
401	C C . .    
402	C C . .    
403	C C . .    
404	C C . .    
405	T T . .    
406	T T . .    
407	G G . .    
408	T T . .    
409	G G . .    
410	C C . .    
411	A A . .    
412	T T . .    
413	A A . .    
414	C C . .    
415	G G . .    
416	G G . .    
417	C C . .    
418	T T . .    
419	G G . .    
420	G G . .    
421	C C . .    
422	T T . .    
423	G G . .    
424	T T . .    
425	A A . .    
426	T T . .    
427	A A . .    
428	T T . .    
429	T T . .    
430	G G . .    
431	C C . .    
432	A G . .  1	 transition  
433	T T . .    
434	C C . .    
435	T A . .  1	 transversion  
436	G G . .    
437	C C . .    
438	T T . .    
439	A A . .    
440	A A . .    
441	A A . .    
442	T T . .    
443	G G . .    
444	T T . .    
445	G G . .    
446	G G . .    
447	T T . .    
448	A A . .    
449	A A . .    
450	C C . .    
451	T T . .    
452	A A . .    
453	G G . .    
454	T T . .    
455	A A . .    
456	A A . .    
457	A A . .    
458	A A . .    
459	G G . .    
460	A A . .    
461	T T . .    
462	G G . .    
463	T T . .    
464	T T . .    
465	G G . .    
466	T T . .    
467	T T . .    
468	A A . .    
469	G G . .    
470	A A . .    
471	T T . .    
472	T T . .    
473	T T . .    
474	T T . .    
475	G G . .    
476	G G . .    
477	G G . .    
478	A A . .    
479	T T . .    
480	C C . .    
481	C C . .    
482	T T . .    
483	T T . .    
484	A A . .    
485	T T . .    
486	A A . .    
487	C C . .    
488	C C . .    
489	T T . .    
490	T T . .    
491	C C . .    
492	A A . .    
493	G G . .    
494	A A . .    
495	T T . .    
496	T T . .    
497	T T . .    
498	G G . .    
499	T T . .    
500	A A . .    
501	T T . .    
502	A A . .    
503	T T . .    
504	T T . .    
505	A A . .    
506	G G . .    
507	C C . .    
508	C C . .    
509	A A . .    
510	A A . .    
511	T T . .    
512	T T . .    
513	T T . .    
514	A A . .    
515	T T . .    
516	C C . .    
517	T T . .    
518	A A . .    
519	C C . .    
520	T T . .    
521	G G . .    
522	A A A .    
523	A A A .    
524	G G G .    
525	G G G .    
526	A A A .    
527	A A A .    
528	G G G .    
529	A A A .    
530	A A A .    
531	A A A .    
532	A A A .    
533	A A A .    
534	A A A .    
535	A A A .    
536	A A A .    
537	T T T .    
538	A A A .    
539	G G G .    
540	C C C .    
541	T T T .    
542	A A A .    
543	C C C .    
544	A A A .    
545	C C C .    
546	T T T .    
547	T T T .    
548	T T T .    
549	T T T .    
550	T T T .    
551	A A A .    
552	T T T .    
553	T T T .    
554	T T T .    
555	G G G .    
556	T T T .    
557	C C C .    
558	A A A .    
559	T T T .    
560	T T T .    
561	G G G .    
562	C C C .    
563	T T T .    
564	A A A .    
565	A A A .    
566	T T T .    
567	A A A .    
568	T T T .    
569	C C C .    
570	T T T .    
571	T T T .    
572	T T T .    
573	T T T .    
574	C C C .    
575	A A A .    
576	G G G .    
577	C C C .    
578	A A A .    
579	C C C .    
580	A A A .    
581	A A A .    
582	G G G .    
583	G G G .    
584	C C C .    
585	A A A .    
586	T T T .    
587	G G G .    
588	T T T .    
589	A A A .    
590	T T T .    
591	A A A .    
592	T T T .    
593	A A A .    
594	C C C .    
595	T T T .    
596	G G G .    
597	T T T .    
598	G G G .    
599	T T T .    
600	A A A .    
601	A A A .    
602	G G G .    
603	T T T .    
604	G G G .    
605	C C C .    
606	A A A .    
607	G G G .    
608	A A A .    
609	A A A .    
610	T T T .    
611	T T T .    
612	T T T .    
613	T T T .    
614	G G G .    
615	G G G .    
616	T T T .    
617	A A A .    
618	A A A .    
619	T T T .    
620	A A A .    
621	C C C .    
622	A A A .    
623	C C C .    
624	A A A .    
625	T T T .    
626	T T T .    
627	T T T .    
628	T T T .    
629	G G G .    
630	A A A .    
631	C C C .    
632	A A A .    
633	A A A .    
634	G G G .    
635	T T T .    
636	T T T .    
637	T T T .    
638	T T T .    
639	C C C .    
640	T T T .    
641	T T T .    
642	C - C .  1	 indel  
643	T - T .  1	 indel  
644	T - T .  1	 indel  
645	T T T .    
646	T T T .    
647	T T T .    
648	T T T .    
649	T T T .    
650	C C C .    
651	T T T .    
652	T T T .    
653	T T T .    
654	G G G .    
655	A A A .    
656	C C C .    
657	C C C .    
658	A A A .    
659	T T T .    
660	T T T .    
661	T T T .    
662	T T T .    
663	C C C .    
664	C C C .    
665	A A A .    
666	A A A .    
667	A A A .    
668	T T T .    
669	C C C .    
670	T T T .    
671	C C C .    
672	A A A .    
673	T T T .    
674	C C C .    
675	T T T .    
676	T T T .    
677	T T T .    
678	A A A .    
679	A A A .    
680	T T T .    
681	T T T .    
682	T T T .    
683	A A A .    
684	T T T .    
685	T T T .    
686	T T T .    
687	G G G .    
688	A A A .    
689	T T T .    
690	T T T .    
691	T T T .    
692	C C C .    
693	A A A .    
694	A A A .    
695	A A A .    
696	A A A .    
697	T T T .    
698	G G G .    
699	A A A .    
700	T T T .    
701	T T T .    
702	T T T .    
703	A A A .    
704	A A A .    
705	T T T .    
706	T T T .    
707	G G G .    
708	A A A .    
709	G G G .    
710	G G G .    
711	A A A .    
712	G G G .    
713	G G G .    
714	A A A .    
715	C C C .    
716	A A A .    
717	C C C .    
718	T T T .    
719	G G G .    
720	T T T .    
721	A A A .    
722	G G G .    
723	T T T .    
724	G G G .    
725	T T T .    
726	T T T .    
727	G G G .    
728	G G G .    
729	T T T .    
730	G G G .    
731	G G G .    
732	C C C .    
733	A A A .    
734	T T T .    
735	G G G .    
736	C C C .    
737	T T T .    
738	G G G .    
739	T T T .    
740	G G G .    
741	C C C .    
742	T T T .    
743	. G G .    
744	. C C .    
745	. A A .    
746	. G G .    
747	. A A .    
748	. T T .    
749	. C C .    
750	. T T .    
751	. G G .    
752	. A A .    
753	. A A .    
754	. A A .    
755	. G G .    
756	. G G .    
757	. T T .    
758	. G G .    
759	. T T .    
760	. T T .    
761	. G G .    
762	. T T .    
763	. T T .    
764	. G G .    
765	. G G .    
766	. . A .    
767	. . G .    
768	. . A .    
769	. . A .    
770	. . A .    
771	. . A .    
772	. . G .    
773	. . A .    
774	. . G .    
775	. . T .    
776	. . G .    
777	. . T .    
778	. . C .    
779	. . A .    
780	. . G .    
781	. . G .    
782	. . G .    
783	. . G .    
784	. . T .    
785	. . C .    
786	. . A .    
787	. . A .    
788	. . T .    
789	. . A .    
790	. . G .    
791	. . G .    
792	. . T .    
793	. . A .    
794	. . A .    
795	. . A .    
796	. . G .    
797	. . T .    
798	. . T .    
799	. . A .    
800	. . G .    
801	. . G .    
802	. . A .    
803	. . A .    
804	. . A .    
805	. . A .    
806	. . C .    
807	. . A .    
808	. . C .    
809	. . A .    
810	. . C .    
811	. . C .    
812	. . A .    
813	. . C .    
814	. . A .    
815	. . C .    
816	. . A .    
817	. . T T    
818	. . T T    
819	. . G G    
820	. . T T    
821	. . T T    
822	. . A A    
823	. . G G    
824	. . C C    
825	. . T T    
826	. . A A    
827	. . G G    
828	. . C C    
829	. . T T    
830	. . C C    
831	. . G G    
832	. . T T    
833	. . T T    
834	. . A A    
835	. . G G    
836	. . C C    
837	. . T T    
838	. . A A    
839	. . G G    
840	. . T T    
841	. . T T    
842	. . T T    
843	. . T T    
844	. . G G    
845	. . T T    
846	. . T T    
847	. . A A    
848	. . G G    
849	. . C C    
850	. . T T    
851	. . A A    
852	. . A A    
853	. . C C    
854	. . T T    
855	. . G G    
856	. . T T    
857	. . T T    
858	. . T T    
859	. . A A    
860	. . G G    
861	. . C C    
862	. . T T    
863	. . A A    
864	. . G G    
865	. . G G    
866	. . C C    
867	. . T T    
868	. . C C    
869	. . A A    
870	. . T T    
871	. . T T    
872	. . A A    
873	. . G G    
874	. . C C    
875	. . T T    
876	. . A A    
877	. . G G    
878	. . C C    
879	. . T T    
880	. . C C    
881	. . C C    
882	. . A A    
883	. . A A    
884	. . C C    
885	. . A A    
886	. . C C    
887	. . C C    
888	. . - N    
889	. . A A    
890	. . C C    
891	. . A A    
892	. . C C    
893	. . G G    
894	. . T T    
895	. . A A    
896	. . G G    
897	. . T T    
898	. . T T    
899	. . A A    
900	. . G G    
901	. . C C    
902	. . T T    
903	. . A A    
904	. . G G    
905	. . C C    
906	. . T T    
907	. . C C    
908	. . G G    
909	. . T T    
910	. . T T    
911	. . A A    
912	. . G G    
913	. . C C    
914	. . T T    
915	. . A A    
916	. . G G    
917	. . C C    
918	. . T T    
919	. . T T    
920	. . T T    
921	. . C C    
922	. . T T    
923	. . T T    
924	. . A A    
925	. . G G    
926	. . C C    
927	. . T T    
928	. . A A    
929	. . A A    
930	. . C C    
931	. . T T    
932	. . G G    
933	. . T T    
934	. . T T    
935	. . T T    
936	. . A A    
937	. . G G    
938	. . C C    
939	. . T T    
940	. . A A    
941	. . G G    
942	. . G G    
943	. . T T    
944	. . T T    
945	. . C C    
946	. . A A    
947	. . T T    
948	. . T T    
949	. . A A    
950	. . G G    
951	. . C C    
952	. . T T    
953	. . A A    
954	. . G G    
955	. . C C    
956	. . T T    
957	. . C C    
958	. . C C    
959	. . A A    
960	. . A A    
961	. . C C    
962	. . A A    
963	. . C C    
964	. . C C    
965	. . A A    
966	. . C C    
967	. . A A    
968	. . C C    
969	. . G G    
970	. . T T    
971	. . A A    
972	. . G G    
973	. . T T    
974	. . T T    
975	. . A A    
976	. . G G    
977	. . C C    
978	. . T T    
979	. . A A    
980	. . G G    
981	. . C C    
982	. . T T    
983	. . C C    
984	. . G G    
985	. . T T    
986	. . T T    
987	. . A A    
988	. . G G    
989	. . C C    
990	. . T T    
991	. . A A    
992	. . G G    
993	. . C C    
994	. . T T    
995	. . T T    
996	. . T T    
997	. . C C    
998	. . T T    
999	. . T T    
1000	. . A A    
1001	. . G G    
1002	. . C C    
1003	. . T T    
1004	. . A A    
1005	. . A A    
1006	. . C C    
1007	. . T T    
1008	. . G G    
1009	. . T T    
1010	. . T T    
1011	. . T T    
1012	. . A A    
1013	. . G G    
1014	. . C C    
1015	. . T T    
1016	. . A A    
1017	. . G G    
1018	. . G G    
1019	. . T T    
1020	. . T T    
1021	. . C C    
1022	. . A A    
1023	. . T T    
1024	. . T T    
1025	. . A A    
1026	. . G G    
1027	. . C C    
1028	. . T T    
1029	. . A A    
1030	. . G G    
1031	. . C C    
1032	. . T T    
1033	. . C C    
1034	. . C C    
1035	. . A A    
1036	. . A A    
1037	. . C C    
1038	. . A A    
1039	. . C C    
1040	. . C C    
1041	. . A A    
1042	. . C C    
1043	. . A A    
1044	. . C C    
1045	. . G G    
1046	. . T T    
1047	. . A A    
1048	. . G G    
1049	. . T T    
1050	. . T T    
1051	. . A A    
1052	. . G G    
1053	. . C C    
1054	. . T T    
1055	. . A A    
1056	. . G G    
1057	. . C C    
1058	. . T T    
1059	. . C C    
1060	. . G G    
1061	. . T T    
1062	. . T T    
1063	. . A A    
1064	. . G G    
1065	. . C C    
1066	. . T T    
1067	. . A A    
1068	. . G G    
1069	. . T T    
1070	. . T T    
1071	. . T T    
1072	. . T T    
1073	. . G G    
1074	. . T T    
1075	. . T T    
1076	. . A A    
1077	. . G G    
1078	. . C C    
1079	. . T T    
1080	. . A A    
1081	. . A A    
1082	. . C C    
1083	. . T T    
1084	. . G G    
1085	. . T T    
1086	. . T T    
1087	. . T T    
1088	. . A A    
1089	. . G G    
1090	. . C C    
1091	. . T T    
1092	. . A A    
1093	. . G G    
1094	. . G G    
1095	. . C C    
1096	. . T T    
1097	. . C C    
1098	. . A A    
1099	. . T T    
1100	. . T T    
1101	. . A A    
1102	. . G G    
1103	. . C C    
1104	. . T T    
1105	. . A A    
1106	. . G G    
1107	. . C C    
1108	. . T T    
1109	. . C C    
1110	. . C C    
1111	. . A A    
1112	. . A A    
1113	. . C C    
1114	. . A A    
1115	. . C C    
1116	. . C C    
1117	. . A A    
1118	. . C C    
1119	. . A A    
1120	. . C C    
1121	. . G G    
1122	. . T T    
1123	. . A A    
1124	. . G G    
1125	. . T T    
1126	. . T T    
1127	. . A A    
1128	. . G G    
1129	. . C C    
1130	. . T T    
1131	. . A A    
1132	. . G G    
1133	. . C C    
1134	. . T T    
1135	. . C C    
1136	. . G G    
1137	. . T T    
1138	. . T T    
1139	. . A A    
1140	. . G G    
1141	. . C C    
1142	. . T T    
1143	. . A A    
1144	. . G G    
1145	. . T T    
1146	. . T T    
1147	. . T T    
1148	. . T T    
1149	. . G G    
1150	. . T T    
1151	. . T T    
1152	. . A A    
1153	. . G G    
1154	. . C C    
1155	. . T T    
1156	. . A A    
1157	. . A A    
1158	. . C C    
1159	. . T T    
1160	. . G G    
1161	. . T T    
1162	. . T T    
1163	. . T T    
1164	. . A A    
1165	. . G G    
1166	. . C C    
1167	. . T T    
1168	. . A A    
1169	. . G G    
1170	. . G G    
1171	. . C C    
1172	. . T T    
1173	. . C C    
1174	. . A A    
1175	. . T T    
1176	. . T T    
1177	. . A A    
1178	. . G G    
1179	. . C C    
1180	. . T T    
1181	. . A A    
1182	. . G G    
1183	. . C C    
1184	. . T T    
1185	. . C C    
1186	. . C C    
1187	. . A A    
1188	. . A A    
1189	. . C C    
1190	. . A A    
1191	. . A A    
1192	. . C C    
1193	. . A A    
1194	. . C C    
1195	. . A A    
1196	. . C C    
1197	. . G G    
1198	. . T T    
1199	. . C C    
1200	. . G G    
1201	. . T T    
1202	. . T T    
1203	. . A A    
1204	. . G G    
1205	. . C C    
1206	. . T T    
1207	. . A A    
1208	. . G G    
1209	. . C C    
1210	. . T T    
1211	. . C C    
1212	. . A A    
1213	. . T T    
1214	. . T T    
1215	. . A A    
1216	. . G G    
1217	. . C C    
1218	. . T T    
1219	. . A A    
1220	. . G G    
1221	. . T T    
1222	. . T T    
1223	. . T T    
1224	. . T T    
1225	. . G G    
1226	. . T T    
1227	. . T T    
1228	. . G A  1	 transition  
1229	. . G G    
1230	. . C C    
1231	. . T T    
1232	. . A A    
1233	. . A A    
1234	. . C C    
1235	. . T T    
1236	. . G G    
1237	. . T T    
1238	. . T T    
1239	. . T T    
1240	. . A A    
1241	. . G G    
1242	. . C C    
1243	. . T T    
1244	. . A A    
1245	. . G G    
1246	. . C C    
1247	. . T T    
1248	. . T T    
1249	. . C C    
1250	. . G G    
1251	. . T T    
1252	. . T T    
1253	. . A A    
1254	. . A A    
1255	. . C C    
1256	. . T T    
1257	. . A A    
1258	. . G G    
1259	. . C C    
1260	. . T T    
1261	. . C C    
1262	. . C C    
1263	. . A A    
1264	. . G G    
1265	. . C C    
1266	. . A A    
1267	. . A A    
1268	. . A A    
1269	. . A A    
1270	. . A A    
1271	. . T T    
1272	. . C C    
1273	. . G G    
1274	. . T T    
1275	. . G G    
1276	. . G G    
1277	. . T T    
1278	. . T T    
1279	. . G G    
1280	. . A A    
1281	. . T T    
1282	. . G G    
1283	. . T T    
1284	. . T T    
1285	. . T T    
1286	. . C C    
1287	. . A A    
1288	. . C C    
1289	. . A A    
1290	. . G G    
1291	. . T T    
1292	. . C C    
1293	. . A A    
1294	. . C C    
1295	. . T T    
1296	. . A A    
1297	. . C C    
1298	. . T T    
1299	. . A A    
1300	. . G G    
1301	. . C C    
1302	. . G G    
1303	. . A A    
1304	. . T T    
1305	. . T T    
1306	. . A A    
1307	. . T T    
1308	. . A A    
1309	. . T T    
1310	. . C C    
1311	. . A A    
1312	. . T T    
1313	. . G G    
1314	. . T T    
1315	. . T T    
1316	. . G G    
1317	. . T T    
1318	. . C C    
1319	. . A A    
1320	. . T T    
1321	. . T T    
1322	. . A A    
1323	. . T T    
1324	. . C C    
1325	. . A A    
1326	. . C C    
1327	. . T T    
1328	. . T T    
1329	. . C C    
1330	. . T T    
1331	. . G G    
1332	. . G G    
1333	. . T T    
1334	. . A A    
1335	. . A A    
1336	. . T T    
1337	. . A A    
1338	. . A A    
1339	. . T T    
1340	. . A A    
1341	. . A A    
1342	. . A A    
1343	. . G G    
1344	. . C C    
1345	. . A A    
1346	. . T T    
1347	. . T T    
1348	. . T T    
1349	. . A A    
1350	. . C C    
1351	. . A A    
1352	. . C C    
1353	. . A A    
1354	. . C C    
1355	. . G G    
1356	. . T .
```
